# Mechanical Considerations of Electrospun Scaffolds for Myocardial Tissue and Regenerative Engineering

**DOI:** 10.3390/bioengineering7040122

**Published:** 2020-10-03

**Authors:** Michael Nguyen-Truong, Yan Vivian Li, Zhijie Wang

**Affiliations:** 1 School of Biomedical Engineering, Colorado State University, Fort Collins, CO 80523, USA; mnguyent@colostate.edu (M.N.-T.); yan.li@colostate.edu (Y.V.L.); 2 Department of Design and Merchandising, Colorado State University, Fort Collins, CO 80523, USA; 3 School of Advanced Materials Discovery, Colorado State University, Fort Collins, CO 80523, USA; 4 Department of Mechanical Engineering, Colorado State University, Fort Collins, CO 80523, USA

**Keywords:** heart failure, left/right ventricle, regenerative therapy, biomechanics, mechanobiology

## Abstract

Biomaterials to facilitate the restoration of cardiac tissue is of emerging importance. While there are many aspects to consider in the design of biomaterials, mechanical properties can be of particular importance in this dynamically remodeling tissue. This review focuses on one specific processing method, electrospinning, that is employed to generate materials with a fibrous microstructure that can be combined with material properties to achieve the desired mechanical behavior. Current methods used to fabricate mechanically relevant micro-/nanofibrous scaffolds, in vivo studies using these scaffolds as therapeutics, and common techniques to characterize the mechanical properties of the scaffolds are covered. We also discuss the discrepancies in the reported elastic modulus for physiological and pathological myocardium in the literature, as well as the emerging area of in vitro mechanobiology studies to investigate the mechanical regulation in cardiac tissue engineering. Lastly, future perspectives and recommendations are offered in order to enhance the understanding of cardiac mechanobiology and foster therapeutic development in myocardial regenerative medicine.

## 1. Introduction

Heart failure is the leading cause of death worldwide and affects about 38 million people [1,2]. There are mainly two types of heart failure, heart failure with reduced ejection fraction (HFrEF) and heart failure with preserved ejection fraction (HFpEF), which involve left ventricular (LV) or right ventricular (RV) or biventricular failures [1]. The pathological remodeling of the myocardium often results in structural and functional changes of the cardiac tissue locally (e.g., in myocardial infarction) or globally (e.g., in idiopathic cardiomyopathy). Currently, pharmaceutical or surgical therapies are not completely satisfactory and fail to halt the continuous deterioration of the myocardium. Consequently, heart transplantation or implantation of a ventricular assist device is the last resort for severe heart failure patients. A preferred treatment is to restore the diseased tissue instead. 

Cardiac tissue and regenerative engineering, via the use of biomaterials with or without cells/molecules to repair heart tissue, is an emerging, interdisciplinary field that aims to improve outcomes and quality of life for these patients [3]. This new field has presented the opportunity to renew and restore the diseased heart [4,5,6]. In order to achieve optimal therapies, the right cell source and the right microenvironment for the cells or their secretome to function are the most important questions to answer. While other reviews have focused on the issues related to the stem/progenitor cells to employ [4,6,7,8], in this review, our main interests lie in the ‘right microenvironment’ for cardiac restoration that is identified or provided by the use of scaffolds. 

The extracellular microenvironment is composed of two aspects: biochemical cues and biophysical cues. The biochemical cues mainly refer to the neighboring cells, soluble factors, extracellular matrix (ECM) proteins, oxygen levels, etc. [9]. The impact of biochemical cues in cardiac restoration has been extensively investigated and reviewed [10,11,12]. The other aspect, the biophysical cues—often referred to as the mechanical environment of the native tissue or a biomaterial (e.g., the elasticity, roughness, surface topology, etc.)—are much less reviewed. It is generally accepted that the mechanical regulation of ECM plays key roles in maintaining tissue homeostasis such as cell proliferation, differentiation, gene/protein expression, and function [11,13,14,15,16,17,18,19,20,21,22]. In this review, we bring attention to the biomechanics of the native myocardium and the microfibrous scaffolds in the consideration of myocardial restoration. We will summarize the development of microfibrous scaffolds in cardiac tissue engineering and their mechanical properties, the current understanding of the cellular responses to mechanical factors (i.e., mechanobiology) using microfibrous scaffolds, and the clinical relevance of the scaffold mechanical properties in myocardial restoration. Finally, we further identify some knowledge gaps to inspire future research and clinical applications of electrospun scaffolds for heart failure patients. 

## 2. Types of Scaffolds in Cardiac Tissue Engineering and Regenerative Medicine

To date, the use of biomaterials in cardiac regenerative research is mainly to (1) serve as an in vitro model system that allows for the mechanistic studies of cardiac and/or progenitor cells to cultivate new treatment strategies; and (2) to be implanted into the myocardium in in vivo models to assist tissue healing. In the latter application, the cardiac scaffolds have been demonstrated to provide mechanical support of the ventricle wall, elicit healing responses, and/or enhance the homing and retention of stem/progenitor cells or molecules in the injured tissue [23,24,25]. Despite different etiologies of heart failure, the majority of regenerative research is limited to myocardial infarction (MI) in the LV as a result of acute or chronic occlusion of coronary arteries [25,26,27]. Recently, there are emerging areas in the restoration of the failing RV associated with pulmonary hypertension (PH) [24]. These preclinical and clinical studies have indicated the potential of scaffolds to restore the damaged myocardium (please see recent reviews [3,4,6,10,28,29,30]). In the past decades, we have gained significant knowledge on the manufacture and use of biomaterials in cardiac regenerative medicine. For instance, it is accepted now that no single biological substance (e.g., fibrin) or synthetic biomaterial (e.g., polyurethane) would likely lead to an optimal therapeutic effect in the MI tissues. Similarly, the delivery of stem/progenitor cells via intravenous or intramyocardial injections alone often results in poor cell retention and cell survival. Therefore, the current trends involve the combined use of a cardiac scaffold (‘cardiac patch’) and regenerative cells or molecules to maximize the repair and healing of ventricles [6,29,30,31,32,33,34]. 

Currently, there are three main resources of cardiac scaffolds: (1) the native polymers found in biological tissues (e.g., collagen, fibrin); (2) the decellularized tissues; (3) the synthetic polymers. Native polymers inspired by the ECM proteins in native tissues are advantageous due to the absence of an immune response, but the lack of biomimetic mechanical behavior has limited the findings and interpretation of data with cells cultured in such non-physiological mechanical conditions. In addition, the synthesis of 3D scaffolds is challenging and research on 3D-printed matrix production remains at the bench stage [3,35]. The second approach, tissue decellularization, offers a quick approach to derive scaffolds with attractive biocompatibility and desired structural and mechanical properties. However, this method is limited by the massive scaffold production with inconsistent qualities from batch to batch, thus preventing a broad use across labs or clinical trials. In contrast to the above two approaches, synthetic polymers offer appropriate mechanical behaviors similar to native tissues and enable ‘off-the-shelf’ production for potential clinical applications. Modifications in the fabrication protocol further enable us to adjust the degradation rates, biocompatibility, porosity, mechanical and conductive properties of the scaffolds. Therefore, in this review, we focus on the microfibrous scaffolds that are fabricated by electrospinning of synthetic materials. 

## 3. Electrospinning of Microfibrous Scaffolds 

Electrospinning is a well-established fiber production method wherein a polymer solution is fed through a high voltage electric field, resulting in coagulation and formation of micro- or nanofibers. The set-up protocols serve bioengineers with the control over the individual fiber size, porosity, alignment, and mechanical properties which are critical in guiding cellular attachment and orientation and eliciting optimal cellular responses [36,37]. For detailed discussions on the methodology of electrospinning in general biomedical applications, please refer to these reviews [29,34,38,39,40,41,42,43,44,45,46,47,48,49,50,51,52]. For reviews specific to cardiac applications, the following reviews are recommended [29,34,39,40,41]. Below, we will only provide a summary of fundamental principles and recent adaptations of electrospinning to cardiac bioengineering applications. 

**Figure 1:**
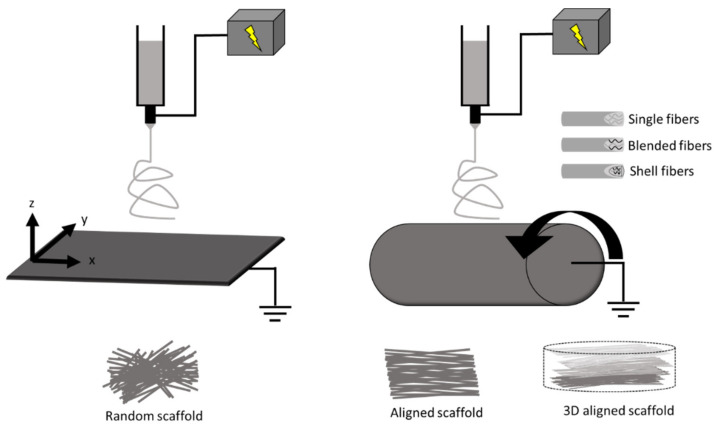
Schematic of electrospinning with a plane (**left**) and a cylinder (**right**) fiber collector, respectively. The movement of collectors (shown by arrows) enables the adjustment of structural properties such as fiber diameter and alignment. Other modifications in the fabrication include blended and core/shell electrospinning to include a hybrid of materials to control the scaffold properties. Scaffolds can also be functionalized, stimulated, or constructed into 3D platforms.

In brief, a polymer solution is ejected through a syringe at a specific flow rate onto a metal collector at a desired distance from the needle tip (Figure 1). A voltage difference is provided between the needle tip and the collector to supply an electric field to “draw out” the polymer fibers. In the production of fibrous sheets, electrospinning is controlled via a variety of parameters in the polymer solution (e.g., molecular weight, concentration) and in the operation of the apparatus (voltage, distance from needle tip to collector plane, injection flow rate, and duration) [6,25,29,38,39,53,54]. These parameters allow for the fine tuning of the chemical (e.g., molecular structure), geometrical or structural (e.g., porosity, fiber diameter, distribution, orientation, morphology), and mechanical properties of the scaffold [38]. 

Some modifications in electrospinning can confer improved properties of the scaffolds. First, the electrospinning process can employ either natural (collagen, silk, cellulose, etc.) or synthetic (polyurethanes, poly(ε-caprolactone), etc.) or a combination of both materials to achieve a variety of structures and utility [29,38,39,55]. These polymers can be combined using either blended or core/shell electrospinning to achieve desired biocompatibility, conductivity, and mechanical strength [56,57,58]. For example, core/shell electrospinning has been used to fabricate a core polymer (poly(lactic acid)/polyaniline) with electroactive property and another shell polymer (poly(lactic acid)/poly(ethylene glycol)) with biocompatible interface [58]. Supporting electrical conductivity is important for synchronous cardiomyocyte contraction in cardiac scaffolds, and similar as well as different fabrication methods have also been explored [59,60]. Second, structural and mechanical properties of scaffolds can be improved by the fabrication process. Typically, a stationary collecting plate allows fibers to be collected in a random manner, whereas a moving plate or rotating mandrel collector is used to create different degrees of aligned fibers [29,36,37,61,62,63,64,65,66] (Figure 1). The fabrication protocol can be adjusted to control scaffold fiber diameter/size, distribution/alignment, porosity, and other physical characteristics. For example, different rotating mandrel speeds could lead to different fiber orientations and anisotropic mechanical properties [63]. Third, modification or treatment of the scaffolds with functional agents (e.g., biomolecules) within or on the fiber surface can improve biological properties. These properties may support cell homing, proliferation, function, differentiation, or survival [27,67,68,69]. For example, matrigel and laminin coatings have been used on electrospun scaffolds to promote cardiomyocyte attachment, morphology, and sarcomere organization [69].

Moreover, the combination of electrospinning with other techniques is able to confer more specific and realistic mechanical properties similar to the native cardiac tissues. For instance, there is a transmural change (100-degree shift) in the myo/collagen fiber orientation from the endocardium to epicardium of the LV [36,70], and such complex 3D anisotropic architecture was achieved in the scaffolds fabricated by electrospinning and laser patterning [71]. In other studies, scaffolds with electrically conductive materials have been explored. Kai et al. presented a blended polypyrrole/poly (ε-caprolactone)/gelatin electrospun scaffolds with the polypyrrole being the driving component for conduction [59]. Moreover, electrospraying of native biomaterial (e.g., decellularized ECM) when combined with electrospinning is an attractive option to better support host cell recruitment while maintaining mechanical support, such as in a cardiac patch [72,73]. Therefore, electrospinning offers the unique capability to fabricate scaffolds mimicking the 3D geometries, mechanical and electrical properties of native myocardial tissues.

## 4. In Vivo Studies: Electrospun Scaffolds in Cardiac Therapies

### 4.1. Cardiac Scaffold as a Mechanical Support 

The use of a cardiac scaffold to treat heart failure patients arose before the emergence of stem cell therapy. It has been found initially that the wrapping of a dilated heart with a biomaterial scaffold could effectively prevent further dilatation, maintain ventricular cavity area, reduce wall stress, and even enhance myocardial function [28,74]. Thus, the early generations of scaffolds were mostly considered to provide mechanical support with acceptable biocompatibility [75]. Currently, the acellular scaffolds are typically in the stiffness range of tens of kPa to tens of MPa and are made of natural or synthetic materials [25,76,77,78,79,80]. 

For instance, the supportive role of cardiac scaffolds is evident in a study using the polyester ether urethane urea (PEEUU) electrospun scaffold with the Young’s modulus of ~1–2 MPa [25]. The PEEUU scaffolds were loaded with adeno-associated viral (AAV) genes and then implanted to the ischemic rat LV. The treatment improved LV function (e.g., increases in ejection fraction and fractional area change). Interestingly, despite this ‘hybrid’ therapeutic approach, the therapeutic effects were found most likely due to the scaffold and not the AAV genes [25]. However, most similar studies did not elaborate how much of the therapeutic effects were from the mechanical support of the scaffold and how much were from the biochemical signals elicited by the scaffolds or delivered cells/genes. In other words, none of the prior studies are designed to investigate the effect of mechanical properties of scaffolds on cardiac restoration. Therefore, the optimal mechanical properties of scaffolds remain unknown. Since the passive mechanical properties of the ventricles are important contributors to the ventricular function [81,82], future investigations should delineate the effects of the scaffold’s mechanical properties to improve the design of cardiac scaffolds. 

### 4.2. Cardiac Scaffold as a Regenerative Support

The current perspective holds that the main mechanisms of scaffold-induced tissue restoration lie in the altered biological functions achieved by the scaffold and/or its delivered biological components, which can more proactively promote the healing of cardiac tissues. Particularly, when loaded with cells or other molecules (e.g., exosomes), the ‘cardiac patch’ enables a more effective induction of remodeling events for tissue renewal. Therefore, the scaffolds should provide a suitable extracellular environment for seeded cellular adhesion, infiltration, and differentiation/growth [24,25,74,83]. Moreover, in order to minimize the invasive delivery of stem/progenitor cells and reduce tumorigenic risks, therapies facilitated with injectable, cell-free ‘cardiac patches’ have recently gained increasing awareness [6,40]. Nevertheless, the ‘match’ of the mechanical property between native myocardium and the ‘cardiac patch’ has not been a consideration in the therapeutic mechanisms. That is, the biological responses to the altered mechanical environment are often ignored in preclinical or clinical studies. 

The lack of the mechanical consideration of cardiac scaffolds is reflected by the variety of Young’s moduli of the scaffolds reported in the literature. Table 1 summarizes the current electrospun scaffolds used in cardiac tissue and regenerative engineering research. It can be seen that the Young’s modulus varies from 20 kPa to 92 MPa, covering sub-physiological and supra-physiological ranges of cardiac tissue elasticity. For instance, Kai et al. showed that a poly(ε-caprolactone)/gelatin patch (with a Young’s modulus of 1.45 MPa), seeded with mesenchymal stem cells (MSCs), improved the angiogenesis and cardiac function in myocardial infarction (MI) rats [74]. In another study, Guex et al. showed that a functionalized MSC-seeded poly(ε-caprolactone) scaffolds (with elastic moduli of 16–18 MPa) stabilized cardiac function and reduced dilatation in rat MI LVs [26]. While these findings are exciting, the therapeutic outcomes are not completely satisfactory and it is difficult to compare these treatments. One of the challenges to interpret and compare the results is due to the ‘random’ selection of scaffold stiffness. As we have noted in the previous Section 4.1, there are a lack of studies on the effects of mechanical properties of scaffolds on therapeutic outcomes. This lack of knowledge further leads to the continuous neglect of this factor in the regenerative treatment, which forms a vicious cycle. Moreover, the scaffold stiffnesses used in the above studies are in orders of magnitude higher than the healthy myocardium, which calls into a question if the cellular performance is impaired by the use of supra-physiologically stiff substrates. Thus, the overall therapeutic outcomes should not only weigh in the multiple aspects of the healing response (angiogenesis, anti-inflammation, anti-oxidant, etc.), but also in the effect of mechanical properties on these healing responses. Additionally, the microstructure and mechanical properties of the substrate are known to form a critical cue to a variety of cells including cardiomyocytes, cardiac myoblasts, and stem/progenitor cells [10,16,84,85]. Overlooking or failing to consider the scaffold’s mechanical impact on tissue remodeling can potentially hamper the development of optimal therapies for heart failure patients. Therefore, it is necessary to explore whether the altered mechanical environment is suitable for the new stem cells or existing cardiac cells to accelerate healing and maximize therapeutic outcomes.

## 5. Mechanical Measurement of Scaffolds

Regardless of the consideration of scaffold mechanical behavior in the study design or not, this physical property is typically reported with one of the following mechanical tests discussed in this section. The most frequently reported mechanical property is the elasticity or stiffness. Furthermore, for implantation purposes, some scaffolds are fabricated to be mechanically similar to the native cardiac tissues. Thus, a proper measurement and comparison of the mechanical properties of scaffolds to those of cardiac tissues is of importance. We summarize the common mechanical testing methods used to characterize the mechanical properties of scaffolds as well as cardiac tissues below. 

Typically, a thin fibrous sheet of scaffold is measured using tensile testing or atomic force microscopy, but these are 2D or 1D mechanical measurements. For cardiac tissues or 3D scaffolds, it is critical to incorporate the planar and transmural mechanical measurements to better characterize the 3D mechanical behavior [96,97,98]. We thus briefly introduce the proper mechanical tests for 3D mechanical measurements. Finally, as the cardiac tissues are viscoelastic, we also include a discussion on the measurement of the material’s dynamic mechanical property—viscoelasticity. 

### 5.1. Elasticity (Young’s Modulus) Measurement

For a linear elastic material, the most important mechanical property is its elasticity, which is often referred to as Young’s modulus (E). Experimentally, the Young’s modulus is a measurement of material’s ability to return to its original shape after a tensile force is applied. Based on this definition, the direct measurement of Young’s modulus is via tensile mechanical tests. It is a fundamental testing method that applies a tensile force (i.e., stress) to a material and then measures the change in deformation (i.e., strain). The Young’s modulus (E) is then defined as the slope of a stress–strain curve. However, native cardiac tissue often presents a nonlinear hyperelastic behavior (see the ‘J-shaped’ stress–strain curve in Table 2), which means that the slope of the stress–strain curve alters at different strains. Such nonlinear, elastic behavior of biological tissues is absent in electrospun scaffolds. Thus, it is important to choose the Young’s modulus (E) at physiological strain ranges to fabricate biomimetic scaffolds. 

Moreover, depending on whether the material is isotropic or anisotropic, uniaxial or biaxial tensile mechanical tests (Table 2) can be performed on the sample to determine E in one or two directions [25,31,54,63,89]. Since an electrospun scaffold is often a thin sheet with identical transmural mechanical behavior, the three-dimensional mechanical measurement is generally not needed. For a randomly aligned electrospun scaffold, the material can be assumed to be isotropic due to the even distribution of the fibers in x and y (planar) directions, and thus a uniaxial tensile test is adequate. But for the aligned scaffold, biaxial tensile testing is more appropriate to simultaneously characterize its anisotropic mechanical behavior [54,63]. The cardiac tissue (myocardium) is well known for its anisotropic mechanical behavior, and thus a better fabrication and mechanical characterization of scaffolds should incorporate multi-axial measurements.

Finally, atomic force microscopy (AFM) is a useful tool for the structural and mechanical measurements of a material (Table 2). A cantilever tip “scans” the surface to obtain high resolution images with topographical characteristics (e.g., roughness) of the material (e.g., scaffold). For mechanical measurement, the cantilever contacts and indents a fiber, and then the force and indentation (deformation/displacement) are measured [99,100]. Because this method is essentially an indentation mechanical test, it is the transverse mechanical property that is directly obtained [99]. To convert the transverse mechanical behavior to the Young’s modulus (assuming isotropic behavior), an axial or planar mechanical property of the “sheet”, different mathematical models are developed and the material is assumed to be isotropic (e.g., the Hertz model is used for isotropic and linear elastic materials) [99]. However, cardiac tissues are orthotropic and nonlinear materials, and electrospun scaffolds are not necessarily isotropic, either. Therefore, the Young’s modulus derived from the AFM measurement may be inaccurate and in fact, it is typically smaller than the modulus directly measured from the tensile mechanical tests [101] (see a further discussion below). Furthermore, the AFM measurement is local and significantly affected by regional variability, and thus multiple measurements in different regions are required to derive a global stiffness. 

### 5.2. Shear Measurement

Sometimes a shear test can be performed to obtain the mechanical property such as shear strength, and the Young’s modulus can be derived indirectly as well (assuming the material is isotropic). While shear testing is not commonly performed on thin scaffolds, its combined use with the biaxial tensile tests is becoming increasingly common to obtain the 3D mechanical property of cardiac tissues, which is orthotropic and exhibits anisotropic shear properties [96,98]. In the development of 3D electrospun scaffolds to better replicate native cardiac tissues, this method should be included to more accurately characterize multi-layered scaffolds. This methodology should also be included in the investigation of the cellular response to a 3D mechanical environment. As shown in Table 2, shear testing is the measurement of an angular deformation of the object when a parallel force is applied to the object’s plane. For cubic specimens, shear testing can provide triaxial shear moduli, which would be useful in the design of orthotropic biomaterials. 

### 5.3. Viscoelasticity Measurement

All the mechanical measurements discussed above are obtained from static mechanical tests (i.e., the response to applied force or deformation is time-independent) and assume the material to be perfectly elastic (i.e., there is no friction energy loss during deformation). However, cardiovascular tissues are viscoelastic materials that experience pulsatile (time-dependent) hemodynamic forces. Therefore, it is imperative to assess the tissue or matrix viscoelastic property that exhibits both viscous and elastic behaviors. 

Viscoelasticity can be measured by applying dynamic mechanical loading in the same mechanical testing system (e.g., tensile tests). The dynamic loading includes cyclic linear (triangle shape) or non-linear (sinusoidal shape) forces applied on the material. Then, the hysteresis area (the area between the loading and unloading stress–strain curves) can be obtained in order to derive the viscoelastic properties. Stress relaxation and creep tests are other traditional methods to measure viscoelasticity [102]. Using a cylindrical geometry of the sample and a sinusoidal compression force applied via a dynamic mechanical analysis (DMA) tester (Table 2), storage modulus, loss modulus, and phase angle can be derived to characterize viscoelastic properties. The storage modulus (E′) measures the energy storage, representing the material’s elasticity, and the loss modulus (E″) measures the energy dissipation, representing the material’s viscosity. Viscosity can also be measured by the material’s damping ratio, the tangent of the E″/E′, or the phase angle, the arctangent of E″/E′ [103,104]. The viscoelastic measurement is not commonly used for mechanical analysis of myocardium or cardiac scaffolds, probably due to the neglect of viscoelastic behavior or the thin sheet geometry (typically about tens or hundreds of µm thickness) that is insufficient for DMA testing (with the thickness of ones of mm). To date, there is only one study that incorporated viscoelasticity into the design of the scaffold. However, this scaffold is made of an ionically crosslinked transparent hydrogel, not by electrospinning [78]. Since the implanted cardiac scaffolds are subjected to pulsatile blood flow, future patches should consider and accommodate for dynamic in vivo loading, and the dynamic mechanical properties should be taken into consideration.

## 6. Discrepant Elastic Moduli Reported from Native Myocardial Tissues in the Literature 

In this review, we would like to point out the important status of discrepant cardiac mechanical data in the current literature. There are a wide variety of reports on the mechanical properties of healthy and diseased myocardium as summarized in Table 3. Indeed, it is true that the elastic modulus of heart tissue varies in different anatomic regions and the stage of injury [78]. However, even under the same condition, the reported values are quite different; for example, the elastic modulus of healthy myocardium ranges from ones of kPa to hundreds of kPa [105,106,107,108,109,110,111]. These inconsistent literature data complicate the selection of appropriate mechanical stiffness for the myocardial scaffold design [111]. 

We noticed that the two most common methods for scaffold mechanical measurements are the AFM (essentially an indentation test) and the tensile tests. It has been well noted that the indentation and tensile mechanical tests generate very different Young’s moduli for the same type of biological tissues (from ones of kPa to hundreds of MPa), with the indentation method consistently yielding lower Young’s moduli [101,111]. This has been supported and thoroughly discussed by McKee et al. [101]. Other factors that may contribute to the inconsistency include the way the tissue is prepared (e.g., solutions used prior and during testing) or mechanically tested (e.g., equibiaxial versus non-equibiaxial testing, maximal strain used) [106,107,108]. While the other factors can be controlled for, the inconsistency due to the intrinsic difference in methodology between the indentation and tensile tests is unavoidable. In any case, the design of cardiac scaffolds requires careful consideration of the myocardium’s mechanical properties (e.g., anatomic region, health status, testing preparation, and methods) for which it replicates. 

As seen in Table 3, depending on the selected ‘modulus’ range, the in vitro experiments may lead to a different conclusion on the mechanobiology of cardiac or stem cells. A few studies have used AFM to derive the mechanical properties of myocardium tissues in small animals (rats, mice, quail) [105,112,113,114]. From these studies, healthy ventricular tissue was reported to have Young’s moduli in the range of ones to tens of kPa. Such mechanical data have been frequently used in the in vitro experimental design as the ‘native myocardium stiffness’ [16,105,114,120]. On the other hand, elastic moduli obtained from tensile mechanical tests are in the range of tens to hundreds of kPa range in the same species [106,108,109]. These values are consistent with measurements in large animal species and humans [96,106,110,117,119]. While most of the prior cardiac tissue engineering studies have adopted the elastic modulus of the matrix as < 60 kPa [16,114,121,122,123,124], the findings on the cellular response should be confirmed in a more physiologically relevant stiffness range. 

## 7. In Vitro Studies: Matrix Mechanics Dependent Cellular Functions in Regenerative Research

The extracellular matrix or scaffold provides a “house” for cells and can regulate the cellular function and behavior via cell–matrix interactions. The mechanical cues with which cells experience are intimately related with the microstructure of the scaffold. A well-known example is that stem cells differentiate into specific lineages (from neurogenic to osteogenic) depending on the relevant mechanical properties of the matrix (from brain to collagenous bone) [16]. Indeed, the influence of matrix mechanics on stem cell behavior or its secreted exosomes has been reported in numerous types of biological tissues. To date, the current cardiac mechanobiology research is performed in a variety of matrix mechanical stiffnesses (macro-scale mechanical measurements). In this section, we will mainly discuss the few mechanobiology studies using electrospun scaffolds (Table 1). In this section, the purpose of our discussion is to highlight the importance of matrix mechanical properties in cellular functions (not limited to progenitor cells), and to raise awareness of the scaffold mechanical properties in future study designs.

To find the optimized chemical and mechanical properties of an electrospun sheet for infarcted myocardial regeneration, Gupta et al. examined the differentiation of embryonic stem cells (ESCs) into cardiomyocytes in different combination of polymers (polyethylene glycol (PEG), poly(ε-caprolactone (PCL), and negatively-charged, carboxylated PCL). Interestingly, they found that it was not the hydrophilic but the elastic property of the scaffold that mostly affected the cardiac differentiation of ESCs. On the softest substrate (4% PEG–86% PCL–10% carboxylated PCL), the ESCs had the highest α-myosin heavy chain expression and intracellular calcium signaling dynamics as well as optimal functional cardiomyocytes [21]. Their data indicated that ESC-derived cardiomyocyte differentiation and maturation can be promoted by tuning the mechanical properties of the polymer scaffold. More importantly, the optimal electrospun scaffolds had a Young’s modulus of 0.71 MPa (compared to others scaffolds of stiffness up to 0.98 MPa). The stiffness range adopted in this study is similar that of the infarcted myocardium and thus the findings are translational in the prediction of regenerative outcomes. 

Another nice experimental study that demonstrated the importance of matrix mechanical properties was the investigation of cellular responses to different 3D scaffolds composed of the same ECM components (decellularized porcine myocardium) [94]. Using different fabrication methods, a decellularized patch, electrospun ECM scaffold, and hydrogel ECM were produced and hMSCs and iPSC-derived cardiomyocytes (iPSC-CMs) were separately cultured on these scaffolds. The ‘stiff’ electrospun scaffold (E = 203 kPa vs. E = 137 kPa from decellularized patch or 0.026 kPa from hydrogel) led to maximal cell viability after 28 days of hMSC culture. Furthermore, the iPSC-CMs presented the maximal expression of connexin-43 when cultured on the ‘stiff’ electrospun scaffolds after 14 days, indicating an enhanced myocyte function. However, the cardiac troponin I expression was minimal in the cells cultured in these scaffolds, indicating a reduced contractile function. While this study strongly advocates for the investigation of the effects of scaffold mechanical properties in cardiac regeneration, similar in vitro research is rarely found. Overall, the cellular response to matrix mechanical properties in the context of cardiac tissue engineering is a largely unexplored area of research, and further investigations using electrospun scaffolds are still warranted.

Other relevant matrix mechanical properties include fiber alignment and 3D structure. The alignment of microfibers has been shown to affect cardiomyocyte behavior [37]. Kai et al. demonstrated that rabbit cardiomyocytes cultured on aligned scaffolds better promoted cell attachment and alignment than those on the randomly aligned scaffolds [88]. Moreover, the design of a 3D scaffold confers the advantage of closely mimicking the orthotropic structure of native myocardium. From such electrospun scaffolds, Wu et al. showed that the 3D structure conferred greater cardiomyocyte alignment, elongation, and functional maturation over a 2D scaffold structure [36]. Therefore, these findings suggest it is critical to include the 3D mechanical property into the scaffold design, with the goal of eliciting a constructive healing response (e.g., anti-inflammatory, angiogenesis, anti-oxidant, etc.) and leading to appropriate cardiac tissue restoration. 

The matrix mechanics, which are measured on a macro-scale, are linked to the microstructure of the matrix with which the cells interact. While biomaterials are commonly designed to mimic the tissue of interest on a macroscale level, the micro- or mesoscales are less considered. Ultimately, the cells interact with the matrix at the micro- or mesoscale, and therefore these smaller scales should also be considered in the design of biomimetic matrices as well [86,125]. D’Amore et al. showed that scaffolds with similar macroscopic biaxial mechanical properties—but different mesoscale topology (i.e., lower fiber intersection density)—resulted in a higher amount of ECM synthesis from smooth muscle cells [125]. This finding was attributed to a change in the cell nuclear aspect ratio. Other studies have developed models that can help to determine the effects of fabrication variables, topology, and geometries on macroscopic mechanical test data using image analysis algorithms alone or in combination with finite element modeling [86,126,127,128]. These efforts are a push to understand materials across multiple scales in order to more closely and comprehensively mimic the native tissues from micro- to macroscale. This consideration in scaffold design will then provide a more precise and accurate control of the mechanobiology in cardiac tissue engineering.

## 8. Are Current Scaffolds Mechanically Biomimetic Enough? 

Besides the lack of consensus of the appropriate physiological mechanical property (i.e., elastic modulus), the neglect of other mechanical factors also hampers the complete understanding of mechanobiology in cardiac tissue engineering. The first limitation is the neglect of the non-linear elastic mechanical behavior of cardiac tissue, and thus only a narrow range of elasticity has been chosen to represent the mechanical environment of the tissue. It is known that the myocardium is a non-linear elastic, anisotropic material [110,116,117]. The full capture of the native tissue’s non-linear elasticity should incorporate a spectrum of mechanical properties (e.g., from systole to diastole) in the design of biomimetic scaffolds. Next, the cellular response has been mostly investigated in a ‘static’ mechanical condition, whereas in physiological conditions the tissue is under cyclic stretch due to the rhythmic heartbeat. To date, only one pioneering study was performed to reveal how cardiomyocytes respond to the dynamic mechanical environment using electrospun silk fibroin scaffolds: it is found that the cyclic stretching (at 10% strain; 1 Hz) along the cell orientation resulted in cardiomyocyte alignment and formation of sarcomeres and gap junctions [64]. Such cellular responses were not observed in cardiomyocytes with the mechanical stimulation perpendicular to the cell orientation. Thus, the consideration of viscoelastic behavior in electrospun scaffolds would advance the understanding of mechanobiology in myocardial tissues. Overall, future studies should consider constructing scaffolds with more realistic mechanical behavior similar to that of native tissue and investigate the mechanobiology of cells under more physiologically relevant mechanical environments. 

## 9. Conclusions and Other Future Perspectives

In this paper, we reviewed the applications of electrospun scaffolds in altering the myocardial healing process to, at least partially, achieve restored functional cardiac tissue. While most prior reviews on electrospun scaffolds focus on the biochemical aspects or fabrication methodologies, we would like to bring attention to the mechanical aspects of the scaffolds in cardiac tissue and regenerative engineering. We briefly go over the electrospinning method, the characterization of mechanical properties with the commonly used methods, and the in vitro and in vivo studies of the application of electrospun scaffolds in cardiac research. We point out the discrepant reports of mechanical properties due to different methodologies (especially between the AFM and tensile mechanical tests), as well as the lack of consensus of the appropriate mechanical properties of the scaffolds to represent the physiological and pathological conditions of the myocardium. Future research should take into consideration the effect of substrate/scaffold mechanical properties on cardiac tissue regeneration. 

In addition to the consideration of mechanical and translational aspects as discussed above, other directions are proposed here as well. Firstly, the fabrication of 3D scaffolds with similar anatomic structure of the cardiac tissue (e.g., helically aligned scaffolds) is suggested, which would allow researchers to create a more realistic in vitro model of the ventricle as both transmural and anatomical regional variations of the fiber orientation can be controlled [35,129,130,131,132]. Second, the design of a more sophisticated and similar physiological mechanical environment is needed. Non-linear elasticity or viscoelasticity of the microfibrous scaffolds have been considered recently but the research is still at the infancy stage. The use of a static mechanical condition in cell culture experiments is not representative of the rhythmic nature of the heart; the dynamic stretch of the scaffold should be included for a more comprehensive study of mechanobiology. Together, the suggested mechanical considerations and other future perspectives will help to strengthen our understanding of cardiac mechanobiology and develop better therapeutics in regenerative medicine.

## Figures and Tables

**Table 1:** Various ranges of the Young’s modulus of electrospun scaffolds used in the cardiac tissue and regenerative engineering studies.

Measurement Method	Material(s)	Young’s Modulus (E)	Summary	Ref.
AFM (individual fiber) and tensile test (sheet)	Polyester urethane urea	7.5 MPa (initial E)	Validation of structural finite element model to examine mechanics of elastomeric fibrous biomaterials with or without smooth muscle cells culture.	[86]
Tensile test	Polyester urethane urea	2.5–2.8 MPa (without smooth muscle cells) 0.3–1.7 MPa (with smooth muscle cells)	Integration of smooth muscle cells into biodegradable elastomer fiber matrix.	[87]
Tensile test	Polypyrrole and poly(ε-caprolactone)/gelatin	8–50 MPa	15 wt% polypyrrole (in 0–30%) exhibited most balanced cardiomyocyte conductivity, mechanical properties, and biodegradability.	[59]
Tensile test	Poly(ε-caprolactone)/gelatin (PG)	1.5 MPa	MSC-seeded PG patch restricted expansion of LV wall, reduced scar size, and promoted angiogenesis.	[74]
Tensile test	Poly(ε-caprolactone) (PCL) and poly(ε-caprolactone)/gelatin (PG)	PCL: Dry: 2–28 MPa Wet: 2–25 MPa PG: Dry: 10–49 MPa Wet: 1–5 MPa	Aligned PG scaffold promoted cardiomyocyte attachment and alignment.	[88]
Tensile test	Gelatin	20 kPa	Construct used to study cardiomyocyte behavior (beating observed) and cardiac proteins expressed for studying cardiac function in drug testing and tissue replacement.	[89]
Tensile test	Polyester urethane urea; polyester ether urethane urea	1–2 MPa	Cardiac patch to deliver viral genes to ischemic rat heart.	[25]
Tensile test	Poly(ε-caprolactone)	16–18 MPa	MSC seeded matrix showed stabilized cardiac function and attenuated dilatation of chronic myocardial infarction in rat.	[26]
Tensile test	Poly(l-lactic acid)-co-poly(ε-caprolactone) (PLACL); poly(l-lactic acid)-co-poly(ε-caprolactone)/collagen (PLACL/collagen)	10–18 MPa	PLACL/collagen scaffold is more suitable compared to PLACL for cardiomyocyte growth and attachment, as well functional activity and protein expression.	[90]
Tensile test	Poly(l-lactide-co-caprolactone) and fibroblast-derived ECM	1–5 MPa	Platform for cardiomyocyte culture and coculture with fibroblasts.	[66]
Tensile test	Polyaniline and poly(lactic-co-glycolic acid)	92 MPa	Development of electrically active scaffold for synchronous cardiomyocyte beating	[91]
Tensile test	Carbon nanotubes embedded aligned poly(glycerol sebacate):gelatin (PG)	93–373 kPa	Contractile properties of cardiomyocytes improved with carbon nanotubes and aligned fibers.	[92]
Tensile test	Polyethylene glycol; polyethylene glycol and poly(ε-caprolactone) (PCL); PCL and carboxylated PCL; polyethylene glycol and PCL and carboxylated PCL	Dry: 18 MPa Wet: 0.7 MPa	Embryonic stem cell derived cardiomyocyte differentiation (α-myosin heavy chain expression, intracellular Ca signaling) is promoted on softer substrates.	[21]
Tensile test	Carbon nanotubes embedded poly(ethylene glycol)-poly(d,l-lactide)	10–60 MPa	Cardiomyocyte protein production and physiological pulse frequency was promoted on core-sheath fibers loaded with 5% carbon nanotubes.	[93]
Tensile test	Digested porcine cardiac ECM and polyethylene oxide	203 kPa	Different rates of cell attachment, survival, and proliferation between ECM patch, electrospun scaffold, and hydrogel.	[94,95]
Tensile test	Reduced graphene oxide modified silk	12–13 MPa	Develop silk biomaterials using controllable surface deposition on nanoscale to recapitulate electrical microenvironments for cardiac tissue engineering.	[60]
Tensile test	Nanofiber yarns	20–110 MPa	3D hybrid scaffold using aligned conductive nanofiber yarns within hydrogel to mimic native cardiac tissue structure induced cardiomyocyte orientation, maturation, and anisotropy, as well as formation of endothelialized myocardium after coculture with endothelial cells.	[36]

**Table 2:** Mechanical testing methods to derive elastic modulus of a material. Young’s modulus = E.

Type	Schematic	Modulus	Methodology
Tensile (upper row: uniaxial test; lower row: biaxial test)	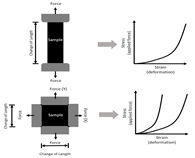	Tensile test: $Young^{\prime }s\;Modulus=\frac{\sigma }{\varepsilon }$ *σ*: stress, *ε*: strain.	1D or 2D tensile (pulling) force applied to a material and the deformation is recorded.
Indentation (in AFM)	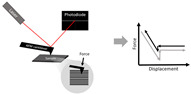	Young’s Modulus derived from a mathematical model. For example, using the Hertz model: $F=\frac{4}{3}\frac{E}{\left(1-v^{2}\right)}\sqrt{r\delta^{3}}$ *δ*: sample indentation, *F*: applied force, *E*: elastic modulus, *v:* Poisson’s ratio *r*: probe tip radius.	The force and indentation (deformation/displacement) are measured from cantilever deflection.
Shear	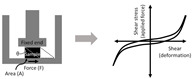	Shear Modulus (*G*) = $\frac{\frac{F}{A}}{\tan\left(\theta \right)}$ For isotropic material, $G=\frac{E}{2\left(1+v\right)}=\frac{E}{3}$ *F*: force, *A*: area, *v*: Poisson’s ratio.	Shear, or parallel frictional force, applied to a material and the change in angle (*θ*) is recorded.
Dynamic Mechanical Analyzer (DMA)	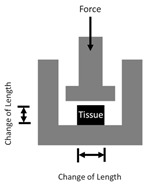	$Storage\;Modulus\;\left(E^{\prime }\right)=\frac{\sigma }{\varepsilon }cos\delta$ $Loss\;Modulus\;\left(E^{\prime \prime }\right)=\frac{\sigma }{\varepsilon }sin\delta$ *σ*: stress, *ε*: strain, *δ*: phase lag between stress and strain.	Oscillatory force applied to a material and resulting displacement is measured.

**Table 3:** Different Young’s moduli reported for the left or right ventricular (LV/RV) tissues. * are data estimated from the original papers. Tissue mechanical property is measured either in main fiber and cross-fiber (X-fiber) directions or in anatomical directions (L: longitudinal (long-axis of ventricle), C: circumferential (short-axis of ventricle)).

Measurement Method	Species/Tissue	Anatomic Region	Young’s Modulus	Ref.
AFM	Mouse/LV	N/A	Embryonic: 12 kPa Neonatal: 39 kPa	[105]
AFM	Rat/LV	Basal surface of tissue section parallel to long axis	Healthy: 18 kPa Infarcted: 55 kPa	[112]
AFM	Mouse/LV	N/A	Healthy: 60 kPa Diseased: 144–295 kPa	[113]
AFM	Quail/Embryonic heart tissue	Apical surface	Healthy: 1–14 kPa	[114]
Custom Indenter	Rat/LV&RV	N/A	Healthy LV: 15 kPa Healthy RV: 13 kPa Hypertensive LV: 12 kPa Hypertensive RV: 22 kPa	[111]
Micropipette aspiration	Rat/Whole heart	N/A	Healthy: Neonatal: 4–11 kPa Adult: 12–46 kPa	[115]
Tensile test	Rat/RV	N/A	Healthy: Low strain (L): 7–18 kPa High strain (L): 464–1054 kPa Low strain (C): 7–17 kPa High strain (C): 421–965 kPa Pressure overloaded: Low strain (L): 18–45 kPa High strain (L): 702–1157 kPa Low strain (C): 5–9 kPa High strain (C): 497–808 kPa	[108]
Tensile test	Rat/RV	Middle of the RV free wall between apex and outflow tract	Healthy: Low strain: 46 kPa High strain: 716 kPa Hypertensive: Low strain: 143 kPa High Strain: 535 kPa	[109]
Tensile test	Rat/LV&RV	N/A	Healthy LV: L: 157 kPa C: 84 kPa Healthy RV: L: 20 kPa C: 54 kPa	[116]
Tensile test	Canine/LV&RV	RV: middle of the free wall; LV: between left anterior descending artery and major marginals of circumflex artery	Healthy LV: Apex-to-base: 125–875 g/cm Circumferential: 250–1375 g/cm Healthy RV: Apex-to-base: 63–1000 g/cm Circumferential: 125–2400 g/cm	[107] *
Tensile test	Canine/LV&RV	RV free wall sinus and conus regions; LV midwall	Healthy RV Sinus: Fiber: 800 g/cm^2^ X-fiber: 500 g/cm^2^ Healthy RV Conus: Fiber: 800 g/cm^2^ X-fiber: 300 g/cm^2^ Healthy LV: Fiber: 600 g/cm^2^ X-fiber: 500 g/cm^2^	[110]
Tensile test	Ovine/LV&RV	Anterior and posterior regions of LV and RV	Healthy LV: Fiber: 113 kPa X-fiber: 23 kPa Healthy RV: Fiber: 100 kPa X-fiber: 40 kPa	[117]*
Tensile test	Ovine/RV	RV free wall	Healthy RV: L: 10–1000 kPa C: 30–2000 kPa Hypertensive RV: L: 80–2000 kPa C: 30–3000 kPa	[118]
Tensile test	Neonatal porcine/LV&RV	Anterior aspect of LV and RV free walls	Healthy LV: Fiber: 10–200 kPa X-fiber: 100–200 kPa Healthy RV: Fiber: 100–200 kPa X-fiber: 50–150 kPa	[119] *
Tensile test	Human/LV&RV	Mid ventricular region of myocardial free wall where muscle structure is uniform	Diseased LV: 70–120 kPa Diseased RV: 80–160 kPa	[106] *
Tensile test	Human/LV, RV, and Septum	N/A	Diseased LV: Fiber: 80–280 kPa X-fiber: 80–160 kPa Diseased Septum: Fiber: 80–320 kPa X-fiber: 40–200 kPa Diseased RV: Fiber: 160–280 kPa X-fiber: 120–240 kPa	[96] *
